# Diethyl 4-(4-ethoxy­phen­yl)-2,6-dimethyl-1,4-dihydro­pyridine-3,5-dicarboxyl­ate

**DOI:** 10.1107/S160053680903339X

**Published:** 2009-08-26

**Authors:** Hoong-Kun Fun, Jia Hao Goh, B. Palakshi Reddy, S. Sarveswari, V. Vijayakumar

**Affiliations:** a X-ray Crystallography Unit, School of Physics, Universiti Sains Malaysia, 11800 USM, Penang, Malaysia; b Organic Chemistry Division, School of Science and Humanities, VIT University, Vellore 632 014, India

## Abstract

In the title compound, C_21_H_27_NO_5_, the dihydropyridine ring adopts a boat conformation. The ethoxy­phenyl ring is oriented approximately perpendicular to the planar part of the dihydropyridine ring, making a dihedral angle of 89.45 (6)°. An intra­molecular C—H⋯O hydrogen bond generates an *S*(6) ring motif. In the crystal structure, neighbouring mol­ecules are linked into chains along the *a* axis by N—H⋯O hydrogen bonds and the chains are inter­connected into two-dimensional networks parallel to the *ab* plane by C—H⋯O hydrogen bonds. The structure is further stabilized by weak C—H⋯π inter­actions.

## Related literature

For general background to and applications of 1,4-dihydro­pyridine derivatives, see: Böcker & Guengerich (1986 ▶); Cooper *et al.* (1992 ▶); Vo *et al.* (1995 ▶); Gaudio *et al.* (1994 ▶); Gordeev *et al.* (1996 ▶); Sunkel *et al.* (1992 ▶). For ring conformations and ring puckering analysis, see: Boeyens (1978 ▶); Cremer & Pople (1975 ▶). For hydrogen-bond motifs, see: Bernstein *et al.* (1995 ▶). For bond-length data, see: Allen *et al.* (1987 ▶). For a related structure, see: Thenmozhi *et al.* (2009 ▶). For the stability of the temperature controller used for the data collection, see: Cosier & Glazer (1986 ▶).
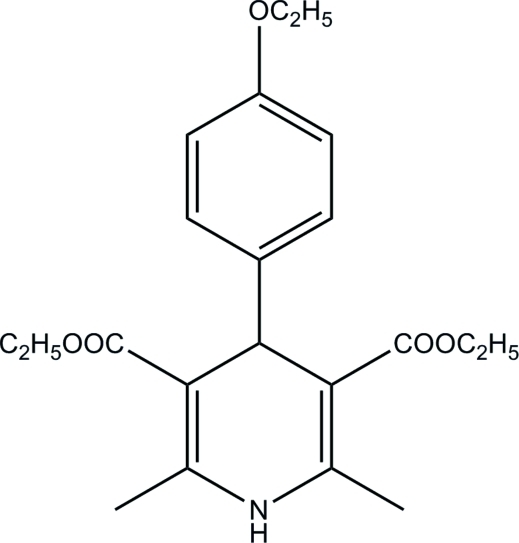


## Experimental

### 

#### Crystal data

C_21_H_27_NO_5_
*M* *_r_* = 373.44
Triclinic, 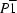

*a* = 7.5557 (1) Å
*b* = 9.5697 (1) Å
*c* = 14.0553 (2) Å
α = 85.844 (1)°
β = 87.679 (1)°
γ = 81.458 (1)°
*V* = 1001.91 (2) Å^3^
*Z* = 2
Mo *K*α radiation
μ = 0.09 mm^−1^
*T* = 296 K
0.28 × 0.27 × 0.07 mm

#### Data collection

Bruker SMART APEXII CCD area-detector diffractometer
Absorption correction: multi-scan (**SADABS**; Bruker, 2005 ▶) *T*_min_ = 0.976, *T*_max_ = 0.994
20664 measured reflections
5290 independent reflections
3602 reflections with *I* > 2σ(*I*)
*R*_int_ = 0.027

#### Refinement

*R*[*F* ^2^ > 2σ(*F* ^2^)] = 0.055
*wR*(*F* ^2^) = 0.161
*S* = 1.02
5290 reflections
253 parameters
H atoms treated by a mixture of independent and constrained refinement
Δρ_max_ = 0.32 e Å^−3^
Δρ_min_ = −0.24 e Å^−3^

### 

Data collection: *APEX2* (Bruker, 2005 ▶); cell refinement: *SAINT* (Bruker, 2005 ▶); data reduction: *SAINT*; program(s) used to solve structure: *SHELXTL* (Sheldrick, 2008 ▶); program(s) used to refine structure: *SHELXTL*; molecular graphics: *SHELXTL*; software used to prepare material for publication: *SHELXTL* and *PLATON* (Spek, 2009 ▶).

## Supplementary Material

Crystal structure: contains datablocks global, I. DOI: 10.1107/S160053680903339X/ci2893sup1.cif

Structure factors: contains datablocks I. DOI: 10.1107/S160053680903339X/ci2893Isup2.hkl

Additional supplementary materials:  crystallographic information; 3D view; checkCIF report

## Figures and Tables

**Table 1:** Hydrogen-bond geometry (Å, °)

*D*—H⋯*A*	*D*—H	H⋯*A*	*D*⋯*A*	*D*—H⋯*A*
N1—H1*N*1⋯O2^i^	0.85 (2)	2.18 (2)	3.0045 (19)	165 (2)
C12—H12*A*⋯O4^ii^	0.97	2.51	3.458 (2)	166
C20—H20*A*⋯O3	0.96	2.14	2.7774 (19)	122
C16—H16*A*⋯*Cg*1^iii^	0.96	2.83	3.767 (2)	165

Symmetry codes: (i) 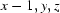; (ii) 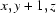; (iii) 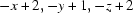. *Cg*1 is the centroid of the C1–C6 benzene ring.

**Fig. 1.**
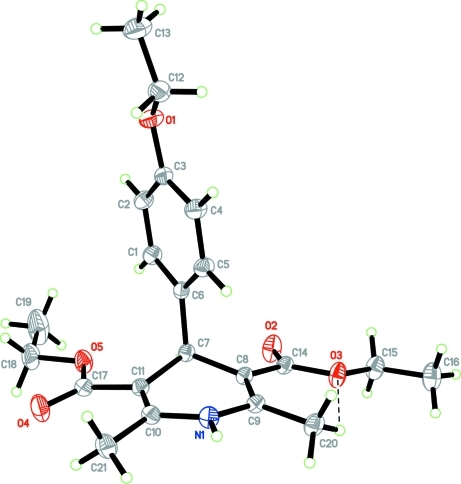
The molecular structure of the title compound, showing 25% probability displacement ellipsoids and the atom-numbering scheme. Hydrogen atoms are shown as spheres of arbitrary radius.

**Fig. 2.**
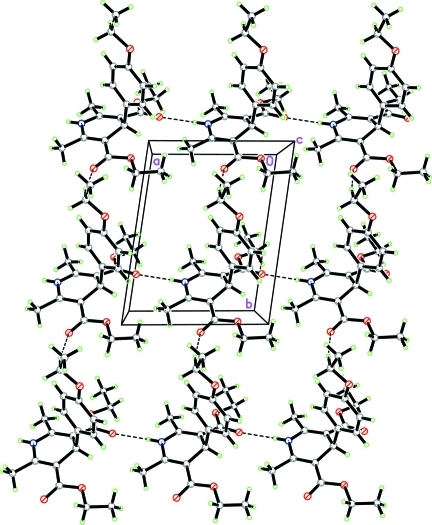
Two-dimensional network parallel to the ab plane, viewed along the c axis. Intermolecular hydrogen bonds are shown as dashed lines.

## supplementary crystallographic information

### Comment

Hantzsch 1,4-dihydropyridines (1,4-DHPS) are biologically active compounds which includes various vasodilator, anti-hypertensive, bronchodilator, heptaprotective, anti-tumor, anti-mutagenic, geroprotective and anti-diabetic agents (Gaudio *et al.*, 1994). Nifedipine, nitrendipine and nimodipine have found commercial utility as calcium channel blockers (Böcker *et al.*, 1986; Gordeev *et al.*, 1996). For the treatment of congestive heart failure, a number of DHP calcium antagonists have been introduced (Sunkel *et al.*, 1992; Vo *et al.*, 1995). Some of DHPs have been introduced as a neuroprotectant and cognition enhancer. In addition, a number of DHPs with platelet anti-aggregatory activity have also been discovered (Cooper *et al.*, 1992).

In the title compound (Fig. 1), the dihydropyridine ring adopts a boat conformation (Boeyens, 1978; Cremer & Pople, 1975) with puckering amplitude Q = 0.2994 (16) Å, θ = 73.0 (3)° and φ = 181.7 (3)°. Atoms C7 and N1 deviate from the C8/C9/C10/C11 plane by 0.362 (2) and 0.143 (2) Å, respectively. The C1-C6 benzene ring is perpendicular to the C8-C11 plane, making a dihedral angle of 89.45 (6)°. An intramolecular C20—H20A···O3 hydrogen bond generates an *S(6)* ring motif (Bernstein *et al.*, 1995). Bond lengths (Allen *et al.*, 1987) and angles are comparable to a related structure (Thenmozhi *et al.*, 2009).

In the crystal structure (Fig. 2), neighbouring molecules are linked into chains along the *a*-axis by N1—H1N1···O2 hydrogen bonds (Table 1). These chains are interconnected into two-dimensional networks parallel to the *ab* plane by C12—H12A···O4 hydrogen bonds. The crystal structure is further stabilized by weak C16—H16A···Cg1 interactions (Table 1).

### Experimental

The title compound was prepared according to Hantzsch pyridine synthesis. A mixture of 4-ethoxybenzaldehyde (10 mmol), ethylacetoacetate (20 mmol) and ammonium acetate (10 mmol) were heated at 353 K for 3 h (monitored by TLC). After completion of the reaction, the mixture was cooled to room temperature and kept for 2 days to get the solid product. The solid formed was washed using diethyl ether. After washing, the solid and the liquid was collected separately and the liquid was kept for solidification. The purity of the crude product was checked through TLC and recrystallized using acetone and ether (m.p. 377–379 K).

### Refinement

Atom H1N1 was located from a difference Fourier map and allowed to refine freely. The other H-atoms were placed in calculated positions, with C-H = 0.93 Å, and *U*_iso_ = 1.2*U*_eq_(C) for aromatic, and C-H = 0.96 Å  and *U*_iso_ = 1.5*U*_eq_(C) for methyl group. A rotating group model was used for the methyl group.

### Crystal data

**Table:**

C_21_H_27_NO_5_	*Z* = 2
*M**_r_* = 373.44	*F*(000) = 400
Triclinic, *P*1	*D*_x_ = 1.238 Mg m^−^^3^
Hall symbol: -P 1	Mo *K*α radiation, λ = 0.71073 Å
*a* = 7.5557 (1) Å	Cell parameters from 5893 reflections
*b* = 9.5697 (1) Å	θ = 2.5–30.2°
*c* = 14.0553 (2) Å	µ = 0.09 mm^−^^1^
α = 85.844 (1)°	*T* = 296 K
β = 87.679 (1)°	Plate, colourless
γ = 81.458 (1)°	0.28 × 0.27 × 0.07 mm
*V* = 1001.91 (2) Å^3^	

### Data collection

**Table:**

Bruker SMART APEXII CCD area-detector diffractometer	5290 independent reflections
Radiation source: fine-focus sealed tube	3602 reflections with *I* > 2σ(*I*)
graphite	*R*_int_ = 0.027
φ and ω scans	θ_max_ = 29.0°, θ_min_ = 2.2°
Absorption correction: multi-scan (*SADABS*; Bruker, 2005)	*h* = −10→10
*T*_min_ = 0.976, *T*_max_ = 0.994	*k* = −11→13
20664 measured reflections	*l* = −19→19

### Refinement

**Table:**

Refinement on *F*^2^	Primary atom site location: structure-invariant direct methods
Least-squares matrix: full	Secondary atom site location: difference Fourier map
*R*[*F*^2^ > 2σ(*F*^2^)] = 0.055	Hydrogen site location: inferred from neighbouring sites
*wR*(*F*^2^) = 0.161	H atoms treated by a mixture of independent and constrained refinement
*S* = 1.02	*w* = 1/[σ^2^(*F*_o_^2^) + (0.0714*P*)^2^ + 0.3075*P*] where *P* = (*F*_o_^2^ + 2*F*_c_^2^)/3
5290 reflections	(Δ/σ)_max_ = 0.001
253 parameters	Δρ_max_ = 0.32 e Å^−^^3^
0 restraints	Δρ_min_ = −0.24 e Å^−^^3^

### Special details

**Table:**

Experimental. The crystal was placed in the cold stream of an Oxford Cyrosystems Cobra open-flow nitrogen cryostat (Cosier & Glazer, 1986) operating at 100.0 (1)K.
Geometry. All esds (except the esd in the dihedral angle between two l.s. planes) are estimated using the full covariance matrix. The cell esds are taken into account individually in the estimation of esds in distances, angles and torsion angles; correlations between esds in cell parameters are only used when they are defined by crystal symmetry. An approximate (isotropic) treatment of cell esds is used for estimating esds involving l.s. planes.
Refinement. Refinement of F^2^ against ALL reflections. The weighted R-factor wR and goodness of fit S are based on F^2^, conventional R-factors R are based on F, with F set to zero for negative F^2^. The threshold expression of F^2^ > 2sigma(F^2^) is used only for calculating R-factors(gt) etc. and is not relevant to the choice of reflections for refinement. R-factors based on F^2^ are statistically about twice as large as those based on F, and R- factors based on ALL data will be even larger.

### Fractional atomic coordinates and isotropic or equivalent isotropic displacement parameters (Å^2^)

**Table:**

	*x*	*y*	*z*	*U*_iso_*/*U*_eq_	
O1	0.73624 (18)	0.59395 (15)	0.53338 (9)	0.0547 (4)	
O2	1.01269 (16)	0.23542 (17)	0.96209 (10)	0.0603 (4)	
O3	0.81388 (15)	0.34092 (15)	1.06441 (9)	0.0511 (3)	
O4	0.5806 (2)	−0.10910 (17)	0.71435 (11)	0.0672 (4)	
O5	0.85133 (17)	−0.04519 (14)	0.72123 (9)	0.0525 (3)	
N1	0.41011 (18)	0.18714 (16)	0.92272 (10)	0.0402 (3)	
C1	0.8807 (2)	0.28106 (19)	0.68730 (11)	0.0416 (4)	
H1A	0.9720	0.2043	0.6870	0.050*	
C2	0.8726 (2)	0.3840 (2)	0.61279 (12)	0.0452 (4)	
H2A	0.9583	0.3761	0.5633	0.054*	
C3	0.7373 (2)	0.49899 (19)	0.61149 (11)	0.0412 (4)	
C4	0.6152 (2)	0.5125 (2)	0.68730 (13)	0.0479 (4)	
H4A	0.5267	0.5911	0.6886	0.058*	
C5	0.6254 (2)	0.40778 (19)	0.76151 (12)	0.0431 (4)	
H5A	0.5421	0.4175	0.8120	0.052*	
C6	0.7553 (2)	0.28953 (17)	0.76285 (10)	0.0333 (3)	
C7	0.7547 (2)	0.16927 (17)	0.84131 (10)	0.0328 (3)	
H7A	0.8750	0.1144	0.8434	0.039*	
C8	0.7073 (2)	0.22640 (17)	0.93892 (10)	0.0330 (3)	
C9	0.5348 (2)	0.24150 (17)	0.97270 (10)	0.0347 (3)	
C10	0.4537 (2)	0.09203 (17)	0.85327 (11)	0.0373 (4)	
C11	0.6234 (2)	0.07125 (17)	0.81710 (10)	0.0352 (3)	
C12	0.5781 (3)	0.6937 (2)	0.51790 (14)	0.0552 (5)	
H12A	0.5585	0.7572	0.5692	0.066*	
H12B	0.4748	0.6447	0.5159	0.066*	
C13	0.6041 (4)	0.7752 (3)	0.42475 (17)	0.0825 (8)	
H13A	0.4990	0.8427	0.4120	0.124*	
H13B	0.6242	0.7112	0.3746	0.124*	
H13C	0.7056	0.8241	0.4278	0.124*	
C14	0.8579 (2)	0.26575 (18)	0.98833 (11)	0.0365 (4)	
C15	0.9594 (2)	0.3755 (2)	1.11817 (13)	0.0532 (5)	
H15A	1.0388	0.4248	1.0765	0.064*	
H15B	1.0280	0.2897	1.1461	0.064*	
C16	0.8798 (3)	0.4675 (3)	1.19452 (16)	0.0695 (6)	
H16A	0.9730	0.4874	1.2336	0.104*	
H16B	0.7964	0.4198	1.2331	0.104*	
H16C	0.8185	0.5546	1.1660	0.104*	
C17	0.6761 (2)	−0.03516 (18)	0.74743 (11)	0.0411 (4)	
C18	0.9092 (3)	−0.1407 (3)	0.64637 (16)	0.0676 (6)	
H18A	0.8361	−0.1144	0.5909	0.081*	
H18B	0.8960	−0.2370	0.6687	0.081*	
C19	1.0976 (4)	−0.1317 (4)	0.6207 (2)	0.1091 (12)	
H19A	1.1366	−0.1932	0.5706	0.164*	
H19B	1.1694	−0.1601	0.6755	0.164*	
H19C	1.1098	−0.0360	0.5993	0.164*	
C20	0.4542 (2)	0.3115 (2)	1.05970 (12)	0.0461 (4)	
H20A	0.5452	0.3102	1.1056	0.069*	
H20B	0.3613	0.2614	1.0870	0.069*	
H20C	0.4046	0.4078	1.0421	0.069*	
C21	0.2974 (2)	0.0239 (2)	0.82690 (14)	0.0506 (5)	
H21A	0.3381	−0.0725	0.8130	0.076*	
H21B	0.2427	0.0748	0.7717	0.076*	
H21C	0.2114	0.0261	0.8792	0.076*	
H1N1	0.303 (3)	0.200 (2)	0.9447 (15)	0.063 (6)*	

### Atomic displacement parameters (Å^2^)

**Table:**

	*U*^11^	*U*^22^	*U*^33^	*U*^12^	*U*^13^	*U*^23^
O1	0.0620 (8)	0.0562 (8)	0.0433 (7)	−0.0086 (7)	0.0050 (6)	0.0103 (6)
O2	0.0287 (6)	0.0946 (11)	0.0613 (8)	−0.0088 (6)	0.0039 (5)	−0.0333 (8)
O3	0.0326 (6)	0.0773 (9)	0.0471 (7)	−0.0096 (6)	−0.0008 (5)	−0.0263 (6)
O4	0.0660 (9)	0.0695 (10)	0.0748 (10)	−0.0262 (8)	0.0099 (7)	−0.0365 (8)
O5	0.0489 (7)	0.0514 (8)	0.0592 (8)	−0.0065 (6)	0.0092 (6)	−0.0238 (6)
N1	0.0265 (7)	0.0497 (9)	0.0451 (8)	−0.0061 (6)	0.0012 (5)	−0.0090 (6)
C1	0.0363 (8)	0.0457 (10)	0.0419 (8)	−0.0037 (7)	0.0077 (7)	−0.0057 (7)
C2	0.0461 (10)	0.0515 (11)	0.0379 (8)	−0.0093 (8)	0.0135 (7)	−0.0056 (7)
C3	0.0469 (9)	0.0434 (10)	0.0352 (8)	−0.0143 (8)	0.0019 (7)	−0.0016 (7)
C4	0.0484 (10)	0.0450 (10)	0.0464 (9)	0.0020 (8)	0.0080 (8)	0.0000 (8)
C5	0.0427 (9)	0.0446 (10)	0.0394 (8)	−0.0015 (7)	0.0118 (7)	−0.0024 (7)
C6	0.0320 (7)	0.0376 (8)	0.0321 (7)	−0.0090 (6)	0.0020 (6)	−0.0075 (6)
C7	0.0282 (7)	0.0370 (8)	0.0326 (7)	−0.0019 (6)	0.0023 (5)	−0.0058 (6)
C8	0.0289 (7)	0.0381 (8)	0.0318 (7)	−0.0037 (6)	0.0001 (5)	−0.0035 (6)
C9	0.0304 (7)	0.0394 (9)	0.0340 (7)	−0.0040 (6)	−0.0003 (6)	−0.0020 (6)
C10	0.0350 (8)	0.0365 (9)	0.0407 (8)	−0.0058 (7)	−0.0051 (6)	−0.0015 (7)
C11	0.0373 (8)	0.0347 (8)	0.0333 (7)	−0.0046 (6)	−0.0018 (6)	−0.0016 (6)
C12	0.0630 (12)	0.0531 (12)	0.0514 (10)	−0.0148 (10)	−0.0137 (9)	0.0034 (9)
C13	0.108 (2)	0.0797 (17)	0.0598 (13)	−0.0203 (15)	−0.0214 (13)	0.0220 (12)
C14	0.0313 (8)	0.0452 (9)	0.0334 (7)	−0.0064 (7)	0.0008 (6)	−0.0042 (6)
C15	0.0381 (9)	0.0764 (14)	0.0486 (10)	−0.0115 (9)	−0.0083 (7)	−0.0171 (9)
C16	0.0631 (13)	0.0843 (17)	0.0649 (13)	−0.0092 (12)	−0.0098 (10)	−0.0308 (12)
C17	0.0466 (9)	0.0382 (9)	0.0387 (8)	−0.0076 (7)	0.0001 (7)	−0.0028 (7)
C18	0.0696 (14)	0.0679 (15)	0.0687 (13)	−0.0092 (11)	0.0135 (11)	−0.0369 (11)
C19	0.0758 (18)	0.135 (3)	0.128 (3)	−0.0290 (18)	0.0381 (17)	−0.086 (2)
C20	0.0338 (8)	0.0614 (12)	0.0430 (9)	−0.0055 (8)	0.0073 (7)	−0.0116 (8)
C21	0.0377 (9)	0.0557 (12)	0.0617 (11)	−0.0132 (8)	−0.0060 (8)	−0.0110 (9)

### Geometric parameters (Å, °)

**Table:**

O1—C3	1.373 (2)	C10—C11	1.352 (2)
O1—C12	1.428 (2)	C10—C21	1.502 (2)
O2—C14	1.2115 (18)	C11—C17	1.465 (2)
O3—C14	1.3353 (19)	C12—C13	1.496 (3)
O3—C15	1.450 (2)	C12—H12A	0.97
O4—C17	1.210 (2)	C12—H12B	0.97
O5—C17	1.351 (2)	C13—H13A	0.96
O5—C18	1.455 (2)	C13—H13B	0.96
N1—C10	1.380 (2)	C13—H13C	0.96
N1—C9	1.380 (2)	C15—C16	1.488 (3)
N1—H1N1	0.85 (2)	C15—H15A	0.97
C1—C2	1.382 (2)	C15—H15B	0.97
C1—C6	1.393 (2)	C16—H16A	0.96
C1—H1A	0.93	C16—H16B	0.96
C2—C3	1.386 (3)	C16—H16C	0.96
C2—H2A	0.93	C18—C19	1.467 (3)
C3—C4	1.382 (2)	C18—H18A	0.97
C4—C5	1.389 (2)	C18—H18B	0.97
C4—H4A	0.93	C19—H19A	0.96
C5—C6	1.383 (2)	C19—H19B	0.96
C5—H5A	0.93	C19—H19C	0.96
C6—C7	1.535 (2)	C20—H20A	0.96
C7—C8	1.523 (2)	C20—H20B	0.96
C7—C11	1.527 (2)	C20—H20C	0.96
C7—H7A	0.98	C21—H21A	0.96
C8—C9	1.360 (2)	C21—H21B	0.96
C8—C14	1.466 (2)	C21—H21C	0.96
C9—C20	1.501 (2)		
			
C3—O1—C12	117.87 (14)	C12—C13—H13A	109.5
C14—O3—C15	117.18 (13)	C12—C13—H13B	109.5
C17—O5—C18	115.21 (15)	H13A—C13—H13B	109.5
C10—N1—C9	123.89 (13)	C12—C13—H13C	109.5
C10—N1—H1N1	118.1 (15)	H13A—C13—H13C	109.5
C9—N1—H1N1	116.6 (15)	H13B—C13—H13C	109.5
C2—C1—C6	121.53 (16)	O2—C14—O3	121.24 (15)
C2—C1—H1A	119.2	O2—C14—C8	123.23 (14)
C6—C1—H1A	119.2	O3—C14—C8	115.52 (13)
C1—C2—C3	120.26 (15)	O3—C15—C16	107.77 (15)
C1—C2—H2A	119.9	O3—C15—H15A	110.2
C3—C2—H2A	119.9	C16—C15—H15A	110.2
O1—C3—C4	124.29 (16)	O3—C15—H15B	110.2
O1—C3—C2	116.40 (14)	C16—C15—H15B	110.2
C4—C3—C2	119.31 (15)	H15A—C15—H15B	108.5
C3—C4—C5	119.52 (16)	C15—C16—H16A	109.5
C3—C4—H4A	120.2	C15—C16—H16B	109.5
C5—C4—H4A	120.2	H16A—C16—H16B	109.5
C6—C5—C4	122.26 (15)	C15—C16—H16C	109.5
C6—C5—H5A	118.9	H16A—C16—H16C	109.5
C4—C5—H5A	118.9	H16B—C16—H16C	109.5
C5—C6—C1	117.05 (15)	O4—C17—O5	120.80 (16)
C5—C6—C7	121.32 (13)	O4—C17—C11	126.77 (16)
C1—C6—C7	121.55 (14)	O5—C17—C11	112.43 (14)
C8—C7—C11	110.43 (12)	O5—C18—C19	108.76 (19)
C8—C7—C6	111.55 (12)	O5—C18—H18A	109.9
C11—C7—C6	109.65 (12)	C19—C18—H18A	109.9
C8—C7—H7A	108.4	O5—C18—H18B	109.9
C11—C7—H7A	108.4	C19—C18—H18B	109.9
C6—C7—H7A	108.4	H18A—C18—H18B	108.3
C9—C8—C14	124.96 (14)	C18—C19—H19A	109.5
C9—C8—C7	120.06 (13)	C18—C19—H19B	109.5
C14—C8—C7	114.94 (12)	H19A—C19—H19B	109.5
C8—C9—N1	118.44 (14)	C18—C19—H19C	109.5
C8—C9—C20	129.12 (15)	H19A—C19—H19C	109.5
N1—C9—C20	112.44 (13)	H19B—C19—H19C	109.5
C11—C10—N1	119.10 (15)	C9—C20—H20A	109.5
C11—C10—C21	128.03 (15)	C9—C20—H20B	109.5
N1—C10—C21	112.87 (14)	H20A—C20—H20B	109.5
C10—C11—C17	120.26 (15)	C9—C20—H20C	109.5
C10—C11—C7	119.61 (14)	H20A—C20—H20C	109.5
C17—C11—C7	119.84 (13)	H20B—C20—H20C	109.5
O1—C12—C13	107.53 (18)	C10—C21—H21A	109.5
O1—C12—H12A	110.2	C10—C21—H21B	109.5
C13—C12—H12A	110.2	H21A—C21—H21B	109.5
O1—C12—H12B	110.2	C10—C21—H21C	109.5
C13—C12—H12B	110.2	H21A—C21—H21C	109.5
H12A—C12—H12B	108.5	H21B—C21—H21C	109.5
			
C6—C1—C2—C3	−0.3 (3)	C9—N1—C10—C11	−14.2 (2)
C12—O1—C3—C4	−15.4 (3)	C9—N1—C10—C21	166.34 (16)
C12—O1—C3—C2	165.31 (16)	N1—C10—C11—C17	176.38 (14)
C1—C2—C3—O1	−178.08 (16)	C21—C10—C11—C17	−4.2 (3)
C1—C2—C3—C4	2.6 (3)	N1—C10—C11—C7	−9.8 (2)
O1—C3—C4—C5	178.09 (17)	C21—C10—C11—C7	169.58 (16)
C2—C3—C4—C5	−2.7 (3)	C8—C7—C11—C10	29.0 (2)
C3—C4—C5—C6	0.4 (3)	C6—C7—C11—C10	−94.32 (16)
C4—C5—C6—C1	1.9 (3)	C8—C7—C11—C17	−157.19 (13)
C4—C5—C6—C7	−174.85 (16)	C6—C7—C11—C17	79.49 (17)
C2—C1—C6—C5	−1.9 (3)	C3—O1—C12—C13	−174.91 (18)
C2—C1—C6—C7	174.78 (16)	C15—O3—C14—O2	−4.4 (3)
C5—C6—C7—C8	−40.9 (2)	C15—O3—C14—C8	176.84 (15)
C1—C6—C7—C8	142.47 (15)	C9—C8—C14—O2	170.76 (18)
C5—C6—C7—C11	81.70 (18)	C7—C8—C14—O2	−11.3 (2)
C1—C6—C7—C11	−94.88 (17)	C9—C8—C14—O3	−10.5 (2)
C11—C7—C8—C9	−28.3 (2)	C7—C8—C14—O3	167.43 (14)
C6—C7—C8—C9	93.93 (17)	C14—O3—C15—C16	175.95 (18)
C11—C7—C8—C14	153.64 (13)	C18—O5—C17—O4	4.9 (3)
C6—C7—C8—C14	−84.16 (16)	C18—O5—C17—C11	−175.20 (16)
C14—C8—C9—N1	−173.78 (15)	C10—C11—C17—O4	2.0 (3)
C7—C8—C9—N1	8.3 (2)	C7—C11—C17—O4	−171.78 (17)
C14—C8—C9—C20	6.4 (3)	C10—C11—C17—O5	−177.96 (15)
C7—C8—C9—C20	−171.48 (16)	C7—C11—C17—O5	8.3 (2)
C10—N1—C9—C8	14.9 (2)	C17—O5—C18—C19	175.6 (2)
C10—N1—C9—C20	−165.23 (15)		

### Hydrogen-bond geometry (Å, °)

**Table:**

*D*—H···*A*	*D*—H	H···*A*	*D*···*A*	*D*—H···*A*
N1—H1N1···O2^i^	0.85 (2)	2.18 (2)	3.0045 (19)	165 (2)
C12—H12A···O4^ii^	0.97	2.51	3.458 (2)	166
C20—H20A···O3	0.96	2.14	2.7774 (19)	122
C16—H16A···Cg1^iii^	0.96	2.83	3.767 (2)	165

Symmetry codes: (i) *x*−1, *y*, *z*; (ii) *x*, *y*+1, *z*; (iii) −*x*+2, −*y*+1, −*z*+2.
